# Chemical-Shift-Encoded Magnetic Resonance Imaging and Spectroscopy to Reveal Immediate and Long-Term Multi-Organs Composition Changes of a 14-Days Periodic Fasting Intervention: A Technological and Case Report

**DOI:** 10.3389/fnut.2019.00005

**Published:** 2019-03-01

**Authors:** Magalie Viallon, Benjamin Leporq, Stephan Drinda, Françoise Wilhelmi de Toledo, Bogdan Galusca, Helene Ratiney, Pierre Croisille

**Affiliations:** ^1^Université de Lyon, Lyon, France; ^2^Centre Hospitalier Universitaire de Saint-Étienne, Saint-Étienne, France; ^3^Université Jean Monnet, Saint-Étienne, France; ^4^CNRS UMR 5520, INSERM U1206, CREATIS, Saint-Étienne, France; ^5^Institut National des Sciences Appliquées de Lyon, Villeurbanne, France; ^6^Klinik St. Katharinental, Diessenhofen, Switzerland; ^7^Buchinger Wilhelmi Clinic, Uberlingen, Germany; ^8^Eating Disorders, Addictions & Extreme Bodyweight Research Group (TAPE) EA, Saint-Étienne, France

**Keywords:** fasting, quantitative imaging, MRI, spectroscopy, chemical shift encoded MRI, quantitative image analysis (QIA), low caloric diet

## Abstract

**Objectives:** The aim of this study was to investigate the feasibility of measuring the effects of a 14-day Periodic Fasting (PF) intervention (<200 cal) on multi-organs of primary interest (liver, visceral/subcutaneous/bone marrow fat, muscle) using non-invasive advanced magnetic resonance spectroscopic (MRS) and imaging (MRI) methods.

**Methods:** One subject participated in a 14-day PF under daily supervision of nurses and specialized physicians, ingesting a highly reduced intake: 200 Kcal/day coupled with active walking and drinking at least 3 L of liquids/day. The fasting was preceded by a 7-day pre-fasting vegetarian period and followed by 14 days of stepwise reintroduction of food. The longitudinal study collected imaging and biological data before the fast, at peak fasting, and 7 days, 1 month, and 4 months after re-feeding. Body fat mass in the trunk, abdomen, and thigh, liver and muscle mass, were respectively computed using advanced MRI and MRS signal modeling. Fat fraction, MRI relativity index T2^*^ and susceptibility (Chi), as well as Fatty acid composition, were calculated at all-time points.

**Results:** A decrease in body weight (BW: −9.5%), quadriceps muscle volume (−3.2%), Subcutaneous and Visceral Adipose Tissue (SAT −34.4%; VAT −20.8%), liver fat fraction (PDFF = 1.4 vs. 2.6 % at baseline) but increase in Spine Bone Marrow adipose tissue (BMAT) associated with a 10% increase in global adiposity fraction (PDFF: 54.4 vs. 50.9%) was observed. Femoral BMAT showed minimal changes compared to spinal level, with a slight decrease (−3.1%). Interestingly, fatty acid (FA) pattern changes differed depending on the AT locations. In muscle, all lipids increased after fasting, with a greater increase of intramyocellular lipid (IMCL: from 2.7 to 6.3 mmol/kg) after fasting compared to extramyocellular lipid (EMCL: from 6.2 to 9.5 mmol/kg) as well as Carnosine (6.9 to 8.1 mmol/kg). Heterogenous and reverse changes were also observed after re-feeding depending on the organ.

**Conclusion:** These results suggest that investigating the effects of a 14-day PF intervention using advanced MRI and MRS is feasible. Quantitative MR indexes are a crucial adjunct to further understanding the effective changes in multiple crucial organs especially liver, spin, and muscle, differences between adipose tissue composition and the interplay that occurs during periodic fasting.

## Background, Motivation, and Objectives

Over much of their evolutionary history, humans faced more physically demanding and/or precarious living conditions and were intermittently challenged by food scarcity. Accordingly, cells and organ systems acquired and retained molecular signaling and metabolic pathways adapted to these environmental challenges, increasing the functionality and resilience of individual cells and the entire organism. As a consequence of the modern hypercaloric or improper diet with a sedentary lifestyle, signaling pathways that mediate the beneficial effects of the responses to these historical environmental challenges for health and disease resistance are disengaged, rendering people vulnerable to obesity, diabetes type 2, cardiovascular disease, cancers, and neurodegenerative disorders. The reversal of the epidemic of diseases caused by unchallenging lifestyles will require a society-wide effort and re-introducing fasting could be a low cost and very efficient therapy intervention (1). Among the various scenarios of intermittent fasting (IF) diets, Periodic Fasting (PF) refers to intermittent fasting regimens with periods of fasting lasting from 2 to 21 or more days (2). To reveal the clinical benefit of such interventions, adequate, robust and ideally non-invasive biomarkers are needed to quantify the impact of PF objectively, study and understand the beneficial and/or eventual adverse effects.

NMR-based quantitative techniques Magnetic Resonance Spectroscopy (MRS) and Imaging (MRI) offer a broad range of approaches that offer unique capabilities to investigate our metabolism and its alterations *in vivo* and non-invasively, that go far beyond the well-known MRI examinations that provide mainly detailed morphological information for diagnosis and clinical use. The power of Magnetic Resonance Spectroscopy (MRS) techniques is in its ability to provide a quantitative metabolic profile targeting a given organ or tissue type (liver, muscle, and bone marrow). To assess, monitor and quantify body fat composition *in vivo*, magnetic resonance spectroscopy (MRS) has been commonly used. ^1^H MRS is used in most of the current MRS protocols, allowing in particular observation and quantification of creatine compounds (skeletal muscles) and intracellular triglycerides (liver and muscles) (3–6). In particular, B-alanyl-L-histidine (carnosine) dipeptide, an essential marker of muscular function and homeostasis, can be assessed (7–10). Phosphorus ^31^P MRS allows for the detection and quantification of several phosphorus-containing metabolites involved in energy and membrane metabolism, which are potentially attractive biomarkers of muscle metabolism (e.g., acetyl-L-carnitine) (11–13).

Along with the biochemical information provided by MRS, quantitative Magnetic Resonance Imaging (MRI) combined with parametric modeling enables the mapping of fat content and the non-invasive measurement and monitoring of changes in body fat distribution. Chemical Shift Imaging (CSI) has recently been used in the musculoskeletal system to assess fat composition in the knee joint and extends MRS with an increasing number of voxels (14). In addition to MRS which suffer from long acquisition times (>4 min per voxel) and does not provide spatial information, quantification of fat can also be performed via Chemical Shift Encoded MRI (CSE-MRI) to separate fat and water in images using the phase variation between fat and water signals at different echo-times (TE) (15). Using multiple echoes acquisition, fat-water separation is feasible using post-processing methods such as IDEAL (Iterative Decomposition of water/fat using Echo Asymmetry and Least-squares estimation) (16). This method has predominantly been used for liver fat assessment and can overcome the MRS gold standard as seen in reference (17) for Proton Density Fat Fraction (PDFF) measurement. CSE-MRI has also been applied to quantify PDFF in the skeleton (18–20), but fatty acid composition itself was not explored in these studies. Recently, CSE-MRI based reconstruction method has been developed to simultaneously quantify PDFF and fatty acid composition *in vitro* and *in vivo* in the fatty liver (21–23), and adipose tissues (24).

The objectives of this pilot study were 1) to acquire preliminary MRI and MRS data in liver, muscle, and bone marrow before, during and after a periodic fasting intervention 2) to show the feasibility of a robust quantitative longitudinal assessment of changes occurring within multiple organs (liver, muscle, bonemarrow) as well as body composition changes with CSE-MRI and MRS 3) to quantify the local and multi-organ impact of such a diet at multiple time points. These are crucial steps to properly design safe and larger longitudinal studies assessing the added value of a joint evaluation of quantitative MR markers and blood samples together for therapeutic intervention.

## Materials and Methods

### Participant Selection

This study was carried out in accordance with the Declaration of Helsinki and the requirements of the French law (articles L1121-1 and L1123-7). The protocol was approved under ID-RCB:2015-A01802-47 by the ethical committee of Lyon Sud-Est II. Participation in the study was voluntary, without monetary compensation except travel/lodging fees, and the experimental procedures, associated risks, and ability to withdraw from the study at any point were explained and documented in a signed informed consent form. The selected volunteer was deemed medically and psychologically healthy based on self-reported health, physical examination by a physician and laboratory testing. Standard exclusion criteria applied were smoking, substance abuse, regular intake of medications, medical or psychiatric illness, and any contraindication to Magnetic resonance imaging (MRI) (e.g., claustrophobia, non-removable metal devices) or known organic comorbidities or current medical treatment, BMI < 30 kg/m^2^.

### Periodic Fasting (PF) Intervention

The fasting intervention protocol was performed according to the guidelines for Buchinger periodic fasting therapy as detailed in (25), and under the supervision of nurses and specialized physicians. Daily clinical visits were planned to monitor the study progress, to support compliance to the intervention including detection of adverse or side effects, and blood chemistry monitoring was performed during the protocol. PF consisted in one “preparation” day with an 800 kcal ovo-lacto-vegetarian diet, followed by 14 pure fasting days, and three days of a progressive ovo-lacto-vegetarian refeeding (starting with 800 kcal/day, with a 200 kcal increment every day). During the pure fasting days, an ad libitum consumption of water and herbal tea was allowed, and it was mandatory to drink ≥3 L of liquids. Breakfast was including 250 ml of tea sweetened with 20 g of honey (= 15 g of carbohydrates); 250 ml of a vegetable broth was served for lunch and dinner for a total caloric intake of 128 Kcal (see details in the Supplemental Table). The subject was asked to perform daily aerobic physical exercise. No medication was used. Criteria for discontinuation of fasting would have been any significant adverse effects including a systolic blood pressure <90 mmHg or a diastolic blood pressure <60 mmHg, a resting heart rate >90 bpm or marked subjective weakness.

### Imaging Protocol and Analysis

All MR imaging (MRI) and spectroscopy (MRS) sessions were acquired on a clinical 3T MRI system (Prisma, Siemens Healthineers, Erlangen, Germany) with the subject in a supine position. For MRI protocol, 2 body-array 18 channel flexible coils were positioned on the liver and upper leg positions and combined with the spine array coil located below the subject. MRS measurements were performed using a dedicate 1H/31P surface coil (Rapid Biomed, Germany). Imaging sessions were performed before at the end of the fasting period, during the building-up period 4 days after refeeding. Long-term acquisitions were planned at 1 month (muscle) or 4 months (abdomen).

#### MRI and MRS Acquisitions

Three MRI acquisitions were sequentially planned for simultaneous quantification of fat content and T2^*^ in the abdomen, tight muscles as well as in the spine and femoral bone marrow*s*: spine and femoral bone marrow adipose tissue (BMAT), visceral (VAT) and subcutaneous (SAT) adipose tissues.

##### Upper and lower abdominal level acquisition protocol

A first transverse acquisition covering the whole abdomen in one breath hold was acquired using a 3D spoiled-multiple echo gradient echo sequence with a flyback readout gradient. The repetition time and flip angle were chosen to avoid T_1_ weighting: TR: 16 ms; Flip-angle: 5°; 12 echoes time (TEs) chosen to emphasize fat and water phase (in/out) at 3T giving an echo train length of *n* = (1:12) × 1.2 ms ; receiver bandwidth = 2000 Hz.pixel^−1^; phase oversampling (slice direction) = 25%; signal averages = 4; FOV = 330–380 mm2 in plane to cover the whole abdomen from liver dome to Sacrum; matrix = 96 × 96 or 128 × 128 ; 32–48 coronal slices; slice thickness = 2–4 mm, scan time: 3–5 min. Phase and magnitude images were systematically saved.

First a 15 s breath-hold (STEAM) T2^*^-corrected single-voxel multi-echo 1H MRS (HISTO; High speed T2-corrected multiple echo 1H MRS-Fat and R2 Quantification, Siemens Healthcare) (26) was performed using stimulated echo acquisition mode, with the following parameters: repetition time = 3,000 ms, echo time (TE) = 12, 24, 36, 48, and 72 ms, 1,024 acquired points, bandwidth = 1,200 Hz, voxel size = 15 × 15 × 15 mm^3^. The acquisition duration for the HISTO sequence was 15 s.

The system will recommend an automatic location for the voxel size based on a 3D gradient-echo e-Dixon vibe (Screening Dixon) sequence (27, 28). Care was taken to verify that it avoided the main liver vessels or gallbladder location. This automatic positioning also maximized the placement of the single-voxel-spectroscopy all through the study. HISTO provides Proton Density Fat Fraction (PDFF) that has been corrected for fat and water transverse relaxation. The PDFF is calculated as the ratio of the methylene resonance area relative to the sum of the water and methylene resonance areas. Since this is a spin echo sequence, R2 values are provided rather than R2^*^ values (R2^*^ is calculated from GRE sequences).

1H MRS liver spectrum was also acquired with a short echo time STEAM sequence (TE = 20 ms), without pre-saturation as described in Coum et al. (29). Main MRS parameters were: TR = 3,000 s, 32 accumulations, with triggering on breathing. From this STEAM–TE20ms a PDFF can also be computed, without T2 correction, with the same ratio, involving methylene and water resonance area as with HISTO. This second measurement of PDFF, with increased accumulations and only one echo time, has been included 1) for quality assessment with a second PDFF measurement to compare with HISTO-PDFF 2) to address settings with very low fat content. For consistency, voxel size and positioning were copied from the HISTO sequence positioning and maintained across all scan sessions. A volume shim was performed over the chosen voxel. Acquisition time for MRS was ~2–4 min.

##### Thigh level acquisition protocol

The patient was then repositioned for the thigh examination in prone position feet first. The subject was placed in the prone position, feet first lying on the surface coil positioned below the quadriceps muscle, centered 15 cm above the upper edge of the patella and wrapped around the leg. A vacuum mattress, molded to the specific contours of the patient's thigh without applying pressure, provided fast, comfortable, and reproducible immobilization for the entire leg of the subject. Under-pressured at first examination to model the thigh of the volunteer, and ensuring a standardized personalized position of the quadriceps femoris muscle at rest, this protocol warranted that subject positioning was reproducible across imaging sessions, with a similar orientation of the muscle fibers to B0 direction.

A 3D spoiled gradient echo sequence was acquired covering the whole leg with 48 transverse slices of 5 mm thick were acquired with a 400 × 280 mm2 in-plane field of view and a 256 × 160 acquisition matrix giving a voxel size of 1.56 × 1.56 × 5 mm^3^. The main MR parameters were TR/Flip angle: 22 ms/5°, receiver bandwidth: 1,395 Hz/pixel, and 1 signal average. TR and flip angle were adjusted to minimize the T1-related bias. Phase and magnitudes images were reconstructed systematically. The total coverage in the z-direction was 240 mm, while analysis coverage focused on 140 mm free from any aliasing artifacts.

A three-dimensional (3D) higher resolution gradient echo sequence was also used to sample two gradient echoes after one RF excitation. Imaging parameters were as follows: repetition time (TR) 11.1 ms, echo time (TE) 1.38/2.4/4.8 ms, flip angle 10 degrees, field of view (FOV), = 300–450, bandwidth 1,040 Hz/pixel, water–fat shift 0.15 pixels. The coronal acquisition covered the whole upper leg from meniscus to hips, with a total scan time of 3 min. The voxel size was 0.78 × 0.78 × 3 mm^3^, the number of slices was 120 resulting in a total coverage in the z-direction of 31 cm and 24 cm prior and after aliasing elimination in the slice direction, and an explored 3D volume of 437.5 × 500 × 208 mm^3^, i.e., an in-plane field of view (FOV) of 437.5 × 500 mm^2^.

We used a single-voxel STEAM localization sequence with the following parameters: repetition time (TR) 2,000 ms; echo time (TE) 20 ms; water pre-saturation; number of excitations 180; 2,048 data points; spectral bandwidth of 3,000 Hz; and a total acquisition time of 6:08 min. Following shimming procedures, the linewidth of the water signal was on average ~13 to 16 Hz for the explored muscle.

#### Post-processing

##### CSE-MRI

Reconstruction of the water and fat images from the acquired 2 pts Dixon high-resolution dataset was performed inline (Syngo software, Siemens Healthcare, Erlangen, Germany) using a Dixon approach enabling four 3D isotropic in-phase, out-phase, fat, and water coronal images to be calculated in-line on the MR scanner, hereafter denoted water (W), fat (F), in-phase (IN), and out-phase(OUT) images, respectively.

**Figure 1:**
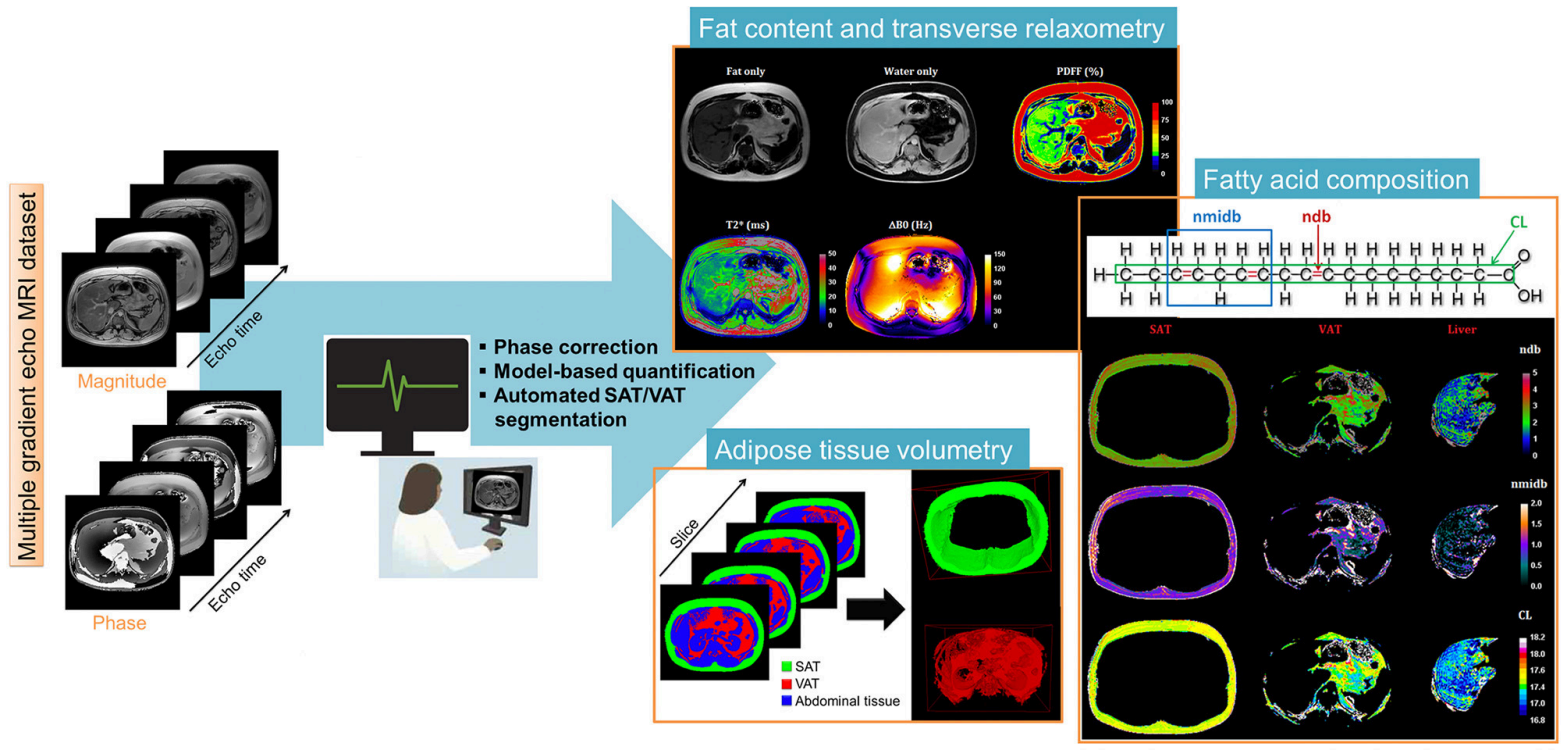
Liver post-processing pipeline of CSE-MRI based analysis. Magnitude and phase images acquired at multiple echo times are processed using an automatized pipeline integrating: phase correction (Phase images were unwrapped to compute the B_0_ field inhomogeneities (ΔB_0_) map and the ΔB_0_-demodulated real part images from which fat-water separation was performed. The fat-water separation step provided parametric ${\text{T}}_{2}^{*}$- and PDFF- maps. From the ΔB_0_ map, external (B_out_), and internal field (B_int_) were separated using the projection unto dipole field. From B_int_, the dipole inversion was performed with a single orientation Bayesian regularization including spatial priors derived from magnitude images for the boundary conditions, error and smoothness weighting to compute the susceptibility map), automated segmentation to derived final Subcutaneous and Visceral Adipose Tissue (SAT/VAT), and cartographies of quantitative parameters PDFF(%), T2^*^(ms), and Chi [~ΔB_0_ (ppm)]. In the adipose tissue, Model-based quantification was performed to extract fatty acid composition. First order radiomic features were extracted for each parametric map.

**Figure 2:**
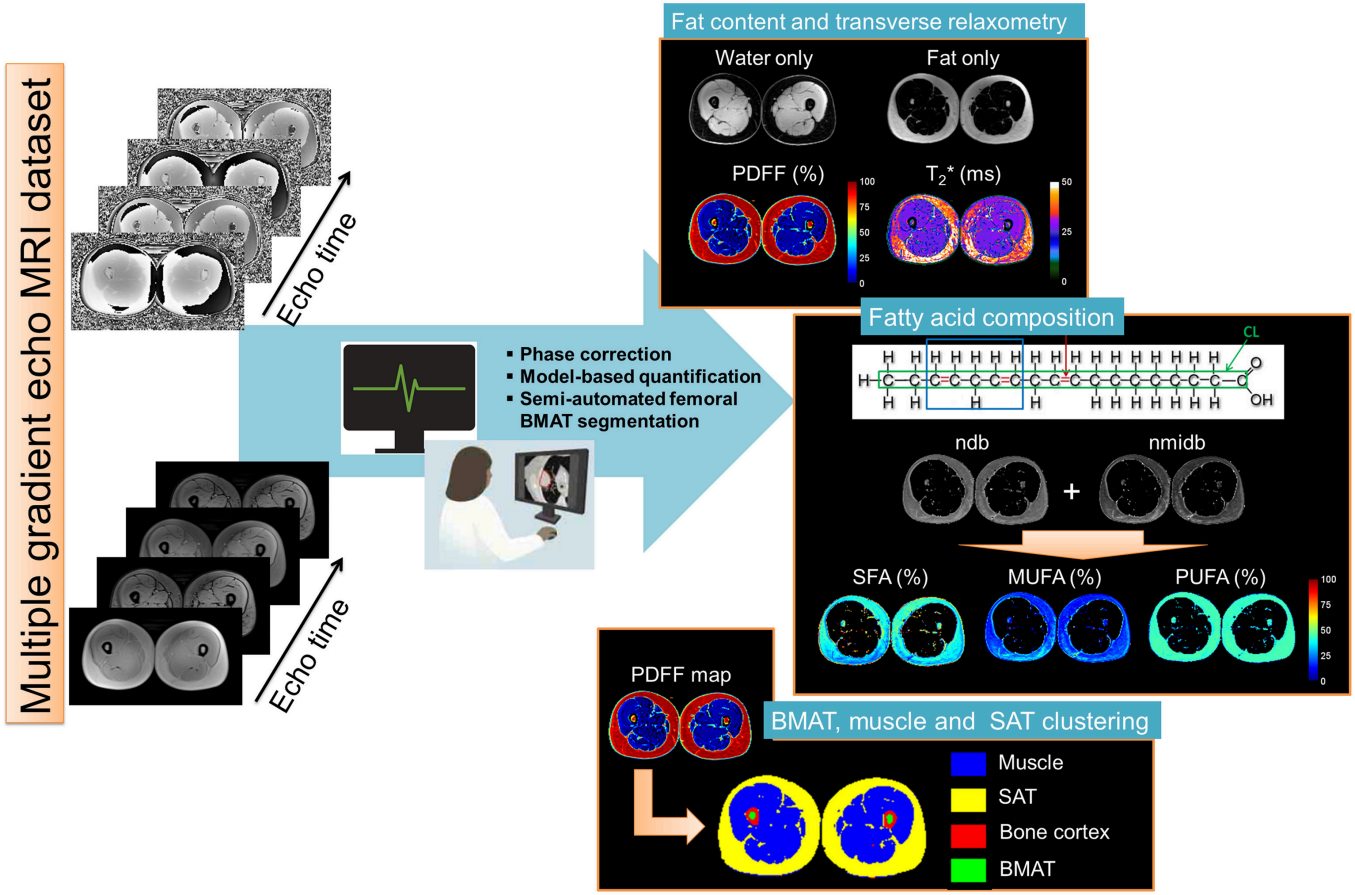
Skeletal muscle post-processing pipeline of CSE-MRI based analysis. Magnitude and phase images acquired at multiple echo times are processed using an automatized pipeline integrating: phase correction, fat-water separation to cartographies of quantitative parameters PDFF(%), T2^*^(ms), and Chi [~ΔB_0_ (ppm)], automated segmentation to derived final Subcutaneous and Bone Marrow Adipose Tissue (SAT/BMAT). In the adipose tissue, Model-based quantification was performed to extract Fatty acid composition. First order radiomic features in the muscle were extracted for each parametric map.

For the fat and T2^*^ quantification using the Chemical Shift-Encoded Magnetic Resonance Imaging, the overall image reconstruction workflow is provided in Figure 1 for the liver and Figure 2 for the muscle. To simultaneously quantify fat content and fatty acid composition, we used the method described in Leporq et al. (21, 30) and implemented in Matlab R2016b (The MathWorks Inc., Natick, MA, USA). Briefly, a phase correction algorithm was used to unwrap and correct the native phase images for zero- and first-order phase and rebuild the B_0_-demodulated real part images. Then, using a model of fat ^1^H MR spectrum integrating eight components (Equation 1), the number of double bonds (ndb) and of methylene-interrupted double bonds (nmidb) was derived voxel by voxel by a step wised data fitting procedure on the real part of the corrected signal (Equation 1).

(1) $$\begin{array}{l} S\left(TE\right)=\text{real }((w\times n_{water}+f\times \sum_{k=1}^{8}n_{k}\left(ndb,CL,nmidb\right)\\ \;\;\times e^{2i\pi f_{k}TE})\times e^{-\frac{TE}{T_{2}^{*}}}) \end{array}$$

S(TE) is the real part of the signal over the echo time TE, ${\text{T}}_{2}^{*}$ is the transversal relaxation time, w and f represent the number of water and triglycerides molecules, n_water_ is the number of protons in a water molecule, and n_k_(ndb, CL,nmidb) represents the number of protons of the k-th component of the fat spectrum expressed according to f ndb, nmidb and CL.

This step provided fat and water only images as well as several parametric maps such as ${\text{T}}_{2}^{*}$, B_0_ field inhomogeneity (ΔB_0_), PDFF and saturated (SFA), monounsaturated (MUFA), and polyunsaturated (PUFA) fatty acid fractions (Figure 1). From nbd and nmidb, fatty acid composition was derived according to the relations:

(2) $$\text{UFA=}\frac{\left(\text{ndb}-\text{nmidb}\right)}{3}\times \text{100 }$$

(3) $$\text{PUFA=}\frac{\left(\text{nmidb}\right)}{\text{3}}\times \text{100}$$

MUFA was calculated as MUFA = UFA-PUFA and SFA as SFA = 100 – UFA.

##### Adipose tissue segmentation

To separately compute MRI metric in SAT and VAT and to measure their volumes, an automated segmentation algorithm was implemented. As a first step, the 3D PDFF map was thresholded (*t* = 50%) to build adipose and non-adipose tissue 3D masks.

From the adipose tissue mask, image filling was performed to delimitate SAT outer border. Next, from the center of the volume, pixel erosion with a 12-pixel squared structural element was done to initiate a localized active contour (31). This latter is employed to delimitate the SAT inner border (or VAT outer border) and was next propagated to adjacent slices.

Spine and femoral bone marrow adipose tissues (BMAT) were manually segmented within Osirix on the calculated fat images.

##### MR spectra analysis

**Figure 3:**
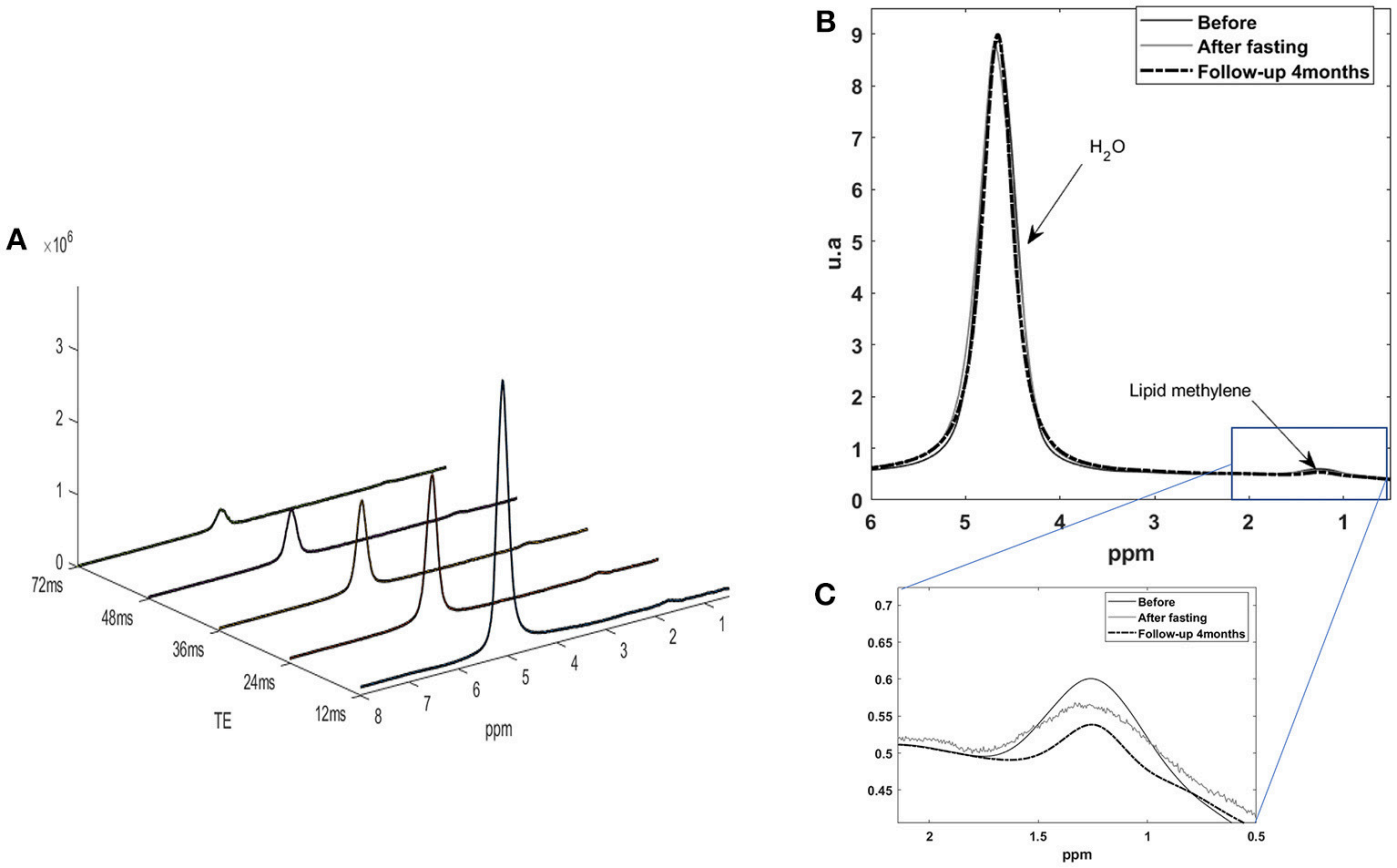
**(A)** Five STEAM monovoxel spectra acquired on the liver with the HISTO sequence before fasting at different TE in order to determine water and lipid T2 and estimate a PDFF corrected for T2 weighting. **(B)** 1H MRS liver spectrum acquired with a short echo time STEAM sequence [here, on echo of the HISTO sequence, TE = 24 ms, NA = 4), without pre-saturation, before, and after fasting. **(C)** Quantification of lipid content for this subject was of 3.3% before and 3.1% after fasting as estimated with QUEST on STEAM –TE20ms (no T2 correction)] and of 3.1 vs. 2.9% for HISTO-PDFF (with T2 correction) estimation, showing consistency among the two measurements.

**Figure 4:**
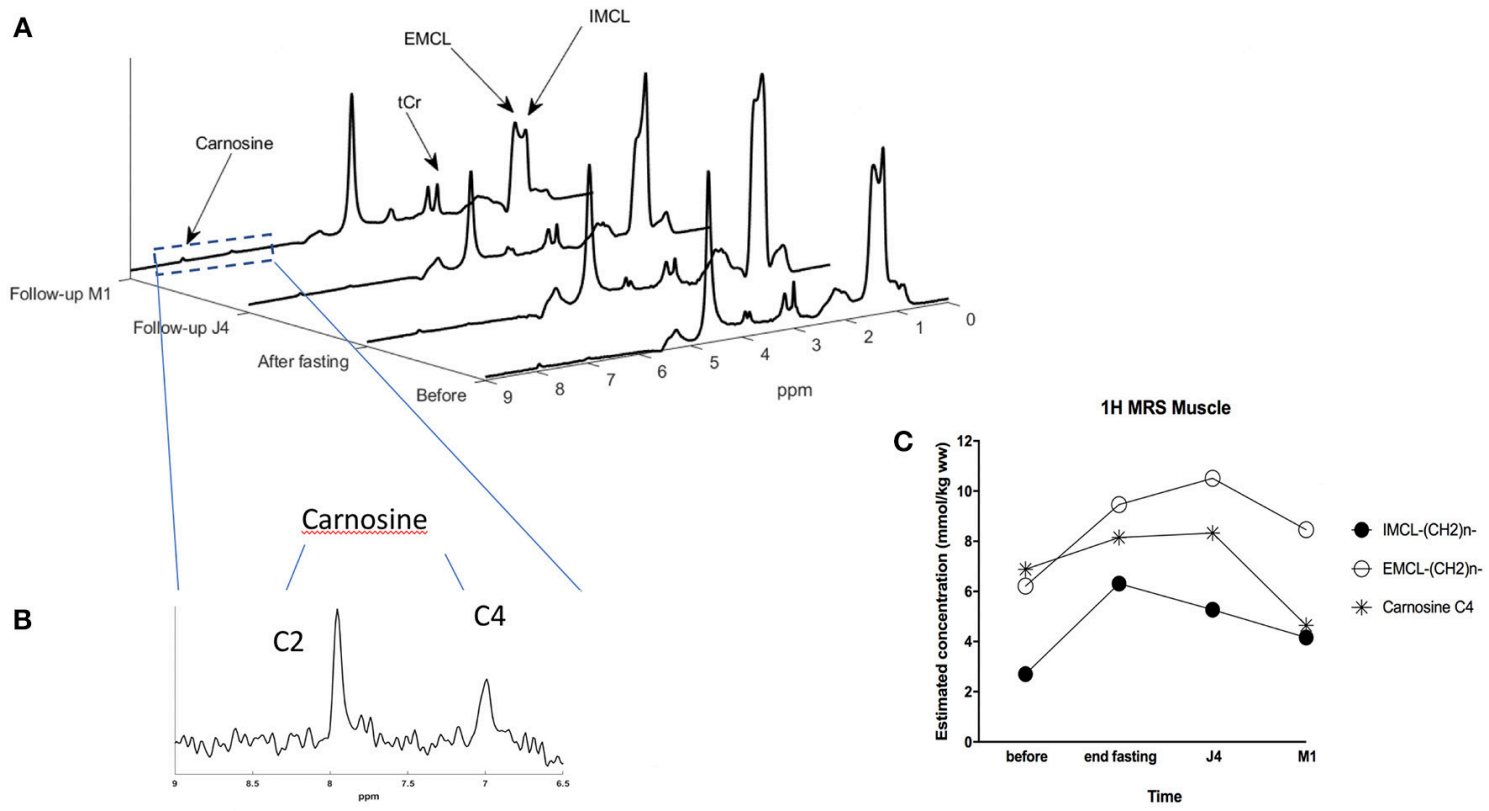
**(A)** STEAM MRS spectra showing the evolution of IMCL (intra-myocellular lipid) and EMCL (extra-myocellular lipid) concentrations in the muscle of the subject at the 4-time points: before and at the end of the fasting period, 4 days after refeeding (build-up period), and 1 month after. **(B)** Zoom on the 6.5 to 9 ppm region providing a detailed view on carnosine C4 and C2 resonances. **(C)** Graph showing the evolution of IMCL, EMCL, and carnosine concentrations in the muscle of the subject at the 4-time points using STEAM MRS monovoxel acquisition.

For the muscle MR spectra, Lorentzian peak definitions, according to Ramadan et al. (32) illustrated in Figure 4a were as follows: 10 lipid peaks, at 0.9 ppm, intramyocellular lipid (IMCL) CH3, at 1.1 ppm, extramyocellular lipid (EMCL)-CH3, at 1.3 ppm IMCL-CH2, at 1.4 and 1.5 ppm EMCL-CH2, a lipid peak at 2 ppm, olefenic fat at 5.2 and 5.3 ppm, creatine CH_3_ peak at 3 ppm trimethylammonium (TMA) at 3.2 ppm; creatine CH2 at, carnosine C2 and C4 at and 8 ppm, respectively (see annotations on spectra in Figures 3, 4).

These Lorentzian peaks were adjusted to actual data according to the time domain quantification method QUEST (33), in its version cQUEST (for customize QUEST) enabling to customize its own prior knowledge constraints on metabolite parameter fitting. It adjusts Lorentzian apodization, frequency shifts compared to prior knowledge picked peaks, and amplitude multiplicative factor Am. Carnosine C4 and C2 peak linewidth were constrained to be the same. For liver spectra, only two lines were used in the fitting procedure, one for the water peak, and one for the lipid peak. Determination of the carnosine and IMCL “absolute” concentration was realized using the creatine signal as internal standard (5) according to the following formula:

(4) $$\begin{array}{l} C_{m}=C_{creatine}\frac{A_{m}}{A_{creatine}}\frac{N_{creatine}}{N_{m}}\\ \;\frac{(1-\exp\left(-TR/T1_{creatine}\right)*\exp\left(-TE/T2_{creatine}\right)}{(1-\exp\left(-TR/T1_{m}\right)*\exp\left(-\frac{TE}{T2_{m}}\right)} \end{array}$$

where *m* is either IMCL-CH2 or EMCL-CH2 or carnosine-C4. Creatine concentration was assumed to be 30 mmol/kg ww; The used relaxation time constants were: T2_creatine_ and T1_creatine_ of Creatine were, respectively 135 ms and 1,000 ms, T2_IMCL/EMCL_ = and T1_IMCL/EMCL_ as found in the literature for human soleus muscle (34), T2_carnosine_ = 152 ms, T1_carnosine_ = 1,488 ms, N is the number of protons of the considered resonating component: N_creatine_ = 3, N_IMCLorEMCL_ = 62, N_carnosine−C4_ = 1

The carnosine content, as well as “intramyocellular” lipid (IMCL) and extramyocellular lipid (EMCL) content, were measured in the skeletal muscles (quadriceps vastus lateralis) of the lower leg by proton magnetic resonance spectroscopy (^1^H- MRS), as previously described (14).

## Results

### Anthropometric and Biological Data

Anthropometric and routine biological data are in the appendix (Table A1). During the fasting period, BW decreased by −7.9 kg (9.5% of total mass). After the refeeding period, BW remained −4.7 kg (−5.7%) at 4 months. These BW changes were associated with a whole quadriceps muscle volume changes of −30mm^3^ (−3.2%) after fasting and −41mm^3^ (−4.4%) after re-feeding. Routine biological sampling showed changes in lipid profile at the end and during follow-up while a transient decrease in leukocytes was observed at the end of fasting (Table 2).

### CSE-MRI

#### Upper and Lower Abdominal Adipose Tissue (SAT/VAT) Analysis

As shown in Table 1, lower abdominal subcutaneous (SAT) and visceral (VAT) adipose tissue volume decreased at the end of fasting intervention, with a more substantial impact on VAT than SAT components (−34.4% VAT vs. −20.8% SAT change), that also translates to a reduction of VAT fraction. After 4 months, SAT and VAT volumes returned near baseline values while VAT volume and VAT fraction remained lower than baseline values. While fatty acid composition in VAT and SAT adipose tissue were remaining quite stable at the end of fasting period, 4 months later, fat composition is showing an increase in saturated FA component in both SAT (52.2 ± 15.6% vs. 46.0 ± 12.6%) and VAT (52.7 ± 19.5% vs. 49.2 ± 17.9%).

**Table 1:** Trunk and lower abdominal adipose tissue analysis.

	**Subcutaneous adipose tissue (SAT)**			**Visceral Adipose Tissue (VAT)**			**Spine bone marrow (BMAT)**		
	**Before**	**After**	**4 months**	**Before**	**After**	**4 months**	**Before**	**After**	**4 months**
Volume (cm^3^)	**650**	**515 (−20.7%)**	**660(+1.5%)**	**491**	**322 (−34.5%)**	**448 (−8.7%)**	45.3	44.6	45
VAT fraction (%)				**43.0**	**38.5 (−10.4%)**	**40.4 (−6%)**			
PDFF (%)	90.9 ± 6.5	92.0 ± 6.4	89.3 ± 6.7	80.6 ± 11.7	80.5 ± 12.0	79.3 ± 11.8	50.9 ± 3.3	54.4 ± 5.6	59.3 ± 1.4
${\text{T}}_{2}^{*}$ (ms)	27.1 ± 18.2	28.0 ± 18.6	25.3 ± 20.2	22.7 ± 17.6	22.5 ± 17.0	22.8 ± 20.5	8.1 ± 3.3	8.2 ± 2.6	7.1 ± 0.2
SFA (%)	44.9 ± 14.3	46.0 ± 12.6	**52.2** **±** **15.6**	48.2 ± 18.7	49.2 ± 17.9	**52.7** **±** **19.5**	**50.9** **±** **6.1**	**42.9** **±** **1.4**	**58.6** **±** **2.1**
MUFA (%)	39.7 ± 10.0	39.8 ± 7.8	36.0 ± 9.0	35.9 ± 11.4	35.9 ± 10.3	33.5 ± 10.8	**36.1** **±** **2.2**	**39.0** **±** **0.5**	**31.7** **±** **1.2**
PUFA (%)	**15.4** **±** **12.4**	**14.2** **±** **9.9**	**11.8** **±** **11.8**	**15.9** **±** **17.2**	**14.9** **±** **15.9**	**13.8** **±** **17.2**	**13.0** **±** **4.0**	**18.1** **±** **1.5**	**9.7** **±** **1.0**

*NB, VAT fraction = VAT/(SAT+VAT)*.

**Table 2:** Liver and upper abdomen adipose tissue analysis.

	**SAT (upper abdomen)**			**VAT (upper abdomen)**			**Liver**		
	**Before**	**After**	**4 months**	**Before**	**After**	**4 months**	**Before**	**After**	**4 month**
VAT fraction **(%)**				**50.7**	**50.5**	**41.4 (−22.4%)**	NA	NA	NA
PDFF (%)	89.0 ± 6.6	87.6 ± 7.7	86.9 ± 7.3	85.2 ± 8.3	84.4 ± 7.8	82.4 ± 8.0	**2.6** **±** **3.3**	**1.4** **±** **3.3**	**3.9** **±** **4.8**
${\text{T}}_{2}^{*}$ (ms)	24.3 ± 15.7	21.4 ± 15.7	22.4 ± 20.8	17.1 ± 17.1	21.1 ± 17.7	16.8 ± 16.8	**15.2** **±** **4.5**	**9.0** **±** **3.1**	**15.4** **±** **5.5**
SFA (%)	**52.4** **±** **10.1**	**44.9** **±** **14.7**	**49.6** **±** **17.5**	**55.2** **±** **19.0**	**50.3** **±** **18.3**	**44.9** **±** **22.4**	NA	NA	NA
MUFA (%)	**37.3** **±** **7.5**	**38.8** **±** **8.3**	**35.8** **±** **9.8**	**32.7** **±** **10.4**	**35.6** **±** **9.8**	**33.2** **±** **12.3**	NA	NA	NA
PUFA (%)	**10.3** **±** **7.3**	**16.3** **±** **14.0**	**14.6** **±** **15.8**	**12.1** **±** **15.8**	**14.1** **±** **14.4**	**21.9** **±** **23.4**	NA	NA	NA

*NB, VAT fraction = VAT/(SAT+VAT)*.

Liver fat fraction decreased at the end of fasting (i.e., PDFF = 1.4 vs. 2.6% at baseline) and re-increased 4-months after fasting (PDFF = 3.9 vs. 1.4%). Unlike in lower abdominal level, upper abdominal SAT and VAT displayed a decreased of saturation after fasting (52.4 vs. 44.9% and 55.2 vs. 50.3% for SAT and VAT, respectively). SAT saturated FA fraction recovered nearly initial value 4 months after fasting (49.6 ± 17.5 vs. 52.4 ±10.1%), while VAT saturated FA fraction continued to decrease in comparison to baseline and end-fasting values (44.9 ± 22.4% M4 vs. 50.3 ± 18.3% baseline), logically paralleled with an increase in unsaturated FA (21.9 ± 23.4 vs. 12.1 ± 15.8%).

Similarly, Table 3 shows that thigh SAT volume decrease after fasting (−7.4%), associated with a slight muscle volume decrease too (−3.3%), similar muscle adiposity, and no significant changes in fatty acid composition.

**Table 3:** Thigh adipose tissue analysis.

	**SAT**			**Femoral BMAT**			**Muscle**		
	**Before**	**After**	**1 month**	**Before**	**After**	**1 month**	**Before**	**After**	**1 month**
Volume (cm^3^)	**470**	**435 (−7.4%)**	**419 (−12.1%)**	278	280 (+0.7%)	279 (+0.3%)	**919**	**889 (−3.3%)**	**878 (−4.4%)**
PDFF (%)	88.0 ± 1.0	87.7 ± 0.4	85.9 ± 1.2	95.8 ± 2.1	92.8 ± 1.7	95.9 ± 1.6	5.43 ± 6.28	5.66 ± 5.99	6.32 ± 6.56
${\text{T}}_{2}^{*}$ (ms)	39.7 ± 3.8	37.4 ± 1.2	38.1 ± 3.9	25.6 ± 3.0	22.7 ± 0.6	25.2 ± 0.5	23.7 ± 6.6	23.4 ± 6.4	23.3 ± 7.1
SFA (%)	52.8 ± 1.1	52.3 ± 0.6	50.0 ± 1.4	**50.0** **±** **2.7**	**49.1** **±** **0.8**	**52.1** **±** **2.2**	NA	NA	NA
MUFA (%)	36.1 ± 0.3	35.9 ± 0.2	36.3 ± 0.7	**38.0** **±** **1.0**	**37.5** **±** **0.7**	**37.8** **±** **1.1**	NA	NA	NA
PUFA (%)	**11.1** **±** **0.8**	**11.8** **±** **0.5**	**13.7** **±** **0.9**	**12.0** **±** **2.1**	**13.4** **±** **0.6**	**10.1** **±** **1.5**	NA	NA	NA

#### Spinal and Femoral Bone Marrow Adipose Tissue (BMAT) Assessment

In the spine, we observed a 10% increase in global adiposity fraction after fasting compared to baseline (Table 1, PDFF: 50.9 vs. 54.4%). 4 months after, the trend was kept constant with PDFF peaking at 59%. At the same time, fatty acid pattern changes were very different compared to the other adipose tissue locations. At the end of fasting, there was a definite decrease of saturated component (−15.7% compared to baseline) while unsaturated components (MUFA + PUFA) were increasing in inverse proportion. After four months, SFA returned to an even higher level than at baseline (58.6 vs. 50.9% at baseline), while unsaturated components MUFA and PUFA reached a lower level than at baseline.

Femoral BMAT (Table 3 Right columns) showed minimal changes compared to spinal level, with a slight decrease (−3.1% change from baseline) after fasting, returning to baseline values at 1 month. PUFA described a similar pattern that observed in spinal level, while SFA was stable at the end of fasting, slightly increasing at 1 month.

### MRS Results

#### Liver

**Figure 5:**
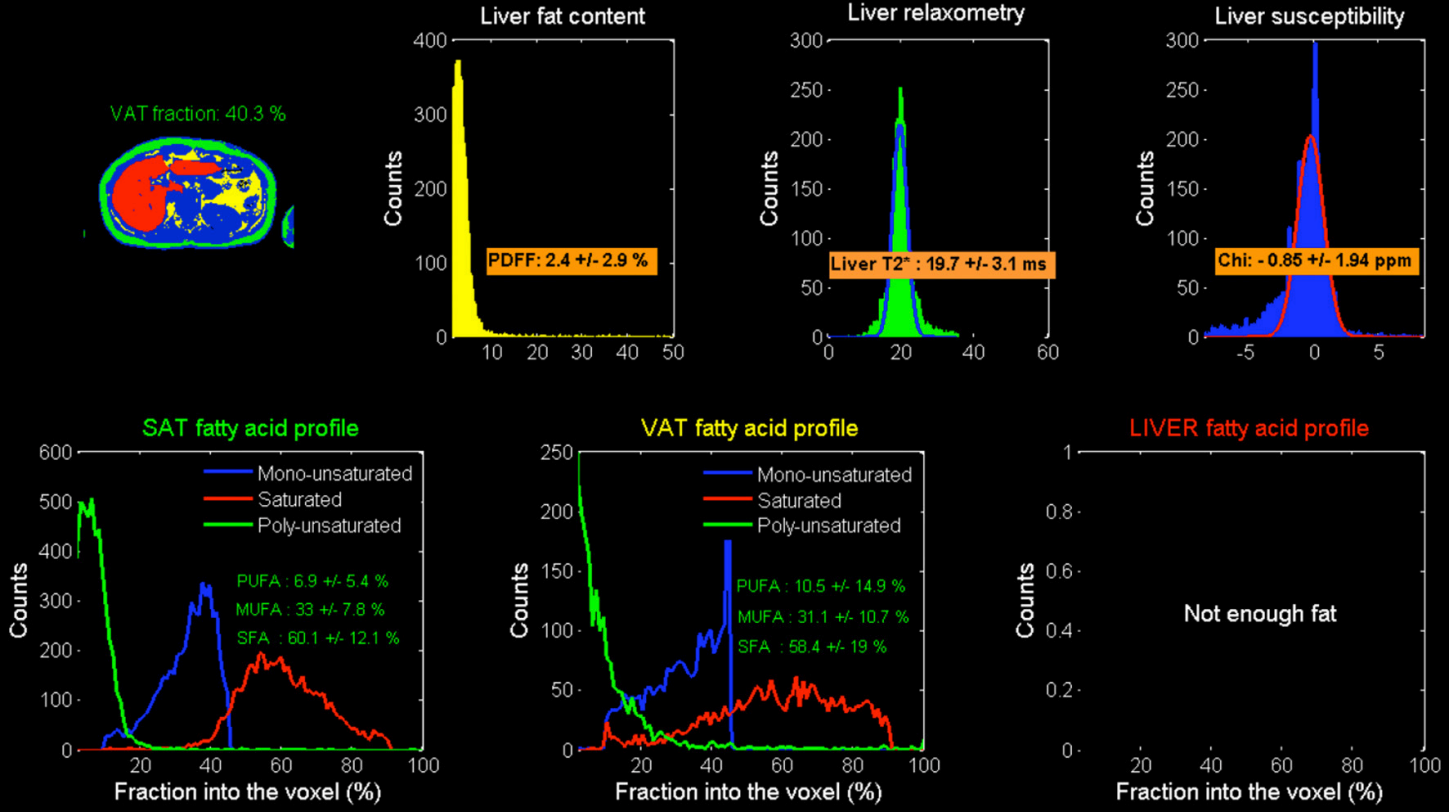
Model-based calculated local Adipose tissue fatty acid composition: results in the liver.

Coherently with CSE-MRI results, HISTO T2-corrected single-voxel multi-echo 1H MRS and STEAM-TE20ms showed a decrease in the liver adiposity after fasting (i.e., PDFF = 3.3 vs. 3% estimated by STEAM TE-20ms and QUEST consistent with HISTO-PDFF = 3.1 vs. 2.9%). Nevertheless, and unlike MRI measurement, MRS monovoxel (both with HISTO and STEAM-TE20ms) continues to show and measure a PDFF decrease 4 months after the end of fasting (PDFF = 2% for both MRS methods) (Figure 3). However, MRI measurement, which provides an average PDFF estimation over the whole liver does not report the same trend, since PDFF increased 4 months after the end of fasting. Figure 5 shows the results of the model-based quantification of SAT/VAT and fatty acid composition in the liver.

#### Skeletal Muscle

Figure 4C shows spectra obtained at baseline, end of fasting, 4 days and 1-month after refeeding, obtained at rest in quadriceps vastus lateralis muscle with each metabolite spectrum as calculated with the QUEST approach.

When focusing on skeletal muscle lipid components changes of extra-myocyte lipids (EMCL) and intra-myocyte lipids (IMCL) components (Figure 4C), and at baseline, there was a near balanced amount of EMCL and IMCL, with a little extra amount of the EMCL. At the end of fasting, all lipids increased, while IMCL increased further compared to EMCL. Figure 4c provides the concentration of IMCL and EMCL (in mmol/kg) showing that IMCL more than doubled after fasting, with an increase from 2.7 at baseline to 6.3 mmol/kg after fasting, and with an incomplete recovery after 1 month (4.16 mmol/kg). In the meantime, EMCL was also increasing from 6.2 to 9.5 mmol/kg while continuing increasing at 10.5 mmol/kg at J4 after refeeding, and incomplete recovery at 1 month.

Carnosine increased from 6.9 at baseline to 8.1 mmol/kg at the end of fasting, remaining increased at J4 (8.3), while returning to lower values after M1 compared to baseline (4.65 mmol/kg) (Figure 4). With this protocol, carnosine precision estimates as determined by the Cramer Rao lower-bound was close to 2%.

## Discussion

We have shown that 3T CSE-MRI enables detailed quantification of both global and organ-specific composition during a fasting intervention. We monitored fat content and fatty acid composition in the fat adipose tissue of central organs of interest during nutrition intervention (liver, bone marrow, muscle, and abdomen). Interestingly, the fat composition changes, as well as its kinetics, are not the same in muscle, liver, and bone marrow, and the interplay between all of these might be of crucial interest

In this case report, we observed that SAT and VAT are still decreased at 4 months with marked polyunsaturated fraction decrease (11.8 vs. 15.4% before fasting) in lower abdominal tissue (11.8 vs. 15.4%) and in the BMAT (10.1% vs. 12 in femoral BMAT and 9.7 vs. 13% in Spine BMAT), whereas in the liver and upper abdominal region SAT a marked increase can be noted (14.6 vs. 10.3%), neither in the thigh SAT (13.7 vs. 11.1%).

This report demonstrates the unique capabilities of non-invasive MRI and ^1^H- MRS approaches to monitor the multi-organ composition changes in a longitudinal PF intervention. This technical report and first findings are based on a single subject longitudinal follow-up study. Accordingly, they will require confirmation with the appropriate study design to explore the impact of low-calorie diets on the distribution of adiposity together with fatty acid composition changes while controlling for the effect of gender, age, and metabolic profiles. These issues are also mattering in obesity since obesity-related disorders are related not only to the total amount of fat, but also to ectopic fat distribution (VAT, IMAT, and liver) and fatty acid composition (35–37). In addition to hepatic lipids, this adipose tissue compartment has also been shown to predict the success or failure of lifestyle intervention with an improvement of insulin sensitivity as a primary marker (38–40).

Indeed muscle and bone are anatomically and functionally intimately connected, with recent evidence highlighting how bone tissue can modulate directly or indirectly skeletal muscle metabolism (41). Hence, we analyzed 2 types of bone marrow: spine bone marrow and femoral bone marrow at the level of quadriceps (~ 10 at ~15 cm from the patella). In each case, the fat adipose tissue in BM has shown significant changes that haven't been observed in SAT and VAT.

It is well established in the literature that women with anorexia nervosa (AN) have increased marrow fat adiposity, despite severe depletion of body fat volume (42). Bredella et al. also evidenced that Women with AN have higher total femoral marrow fat but similar composition compared to normal-weight controls. They also found that the degree of marrow FA unsaturation correlates positively with soleus unsaturation, suggesting that the same factors may influence marrow fat composition as ectopic lipid composition in muscle.

Bone is indeed composed of two tissues: a mineralized osseous component (cortical and trabecular bone) and a marrow component (hematopoietic and fatty marrow). There is substantial evidence that bone marrow adipose tissue (BMAT) is not an inert filler to occupy space, but is a dynamic endocrine organ with multiple functions since intimately involved in bone remodeling, hematopoietic, immune cell differentiation, and energy metabolism (43–45). BMAT is heterogeneous regarding its anatomic distribution (e.g., end-of-bone vs. diaphysis, sub-regionally within the end-of-bone), its activity (constitutive vs. regulated), and its composition (saturated vs. unsaturated fat) (45). Finally, the marrow space is the home of mesenchymal stem cells, which differentiate into either adipocytes or osteoblasts. Saturated fatty acids produced by BMAT adipocytes can blunt the proliferation of mesenchymal stem cells (46) and have lipotoxic effects on osteoblasts and osteoblast differentiation (47–49). Marrow adiposity increases ~7% per decade in the lumbar spine from age 30 to age 80 (50).

The majority of prior MRI studies have evaluated bone marrow fat quantity and composition in the spine; however, a few prior studies have used MRS to study bone marrow fat composition in the hip (18, 51, 52). Pansini et al. (52) performed a sub-regional 3T MRS evaluation of fat content within the greater trochanter, femoral head, femoral neck, and diaphysis of the proximal femur in 80 healthy subjects. They found that total fat content and the conversion index calculated based on total fat content increased with age in both men and women. They did not evaluate saturated and unsaturated fat as in the current study. Martel et al. also found that polyunsaturated fat to be lower (reduced 40–58%) and saturated fat to be higher (+ 13 to + 20%) in postmenopausal women compared to premenopausal women (24, 53).

While prior studies have used 1H MRS to analyze fat composition in the spine only, we demonstrated here that CSE-MRI is a promising alternative approach to investigate, within a multi-organ approach, both femoral bone and spine marrow adipose tissue composition. Previously, bone marrow fat composition analysis was only possible by using ^1^H MRS, which also translates to extended scan times to evaluate multiple sub-regions. In this study, the scan duration was ~4–6 min/targeted organ, which is a suitable time in a clinical context. Such estimation is made assuming mono-exponential behavior for water and triglycerides resonances, which is a valid assumption considering the short train echo length used minimizing the T2^*^ weighting, the preponderance of MAT in this tissue and fieldmap corrections applied before quantification (54, 55). The described CSE-MRI method has been first validated *in-vitro* and evaluated in volunteers while showing that the error in FA composition estimation of this approach was minimal. The robustness of the model function has been recently evaluated and compared against other model variants from both theoretical and experimental conditions perspectives. Using *in vitro* and *in vivo* experimental settings with ^1^H MRS set as the reference and including test-retest variability assessment, authors also showed that the proposed function model was robust for FA composition assessment in human volunteers. In another recent study, both NMR approaches (CSE-MRI and 1H-MRS) were compared to gas chromatography-mass spectroscopy (GS-MS), the ground-truth technique for FA quantification in tissue samples. Both NMR approaches were found well correlated with GS-MS results, and CSE-MRI sequence associated with the dedicated post-processing was found reliable for longitudinal studies. FA indexes proposed in the current study have been showed to be close to absolute values obtained by GS-MS.

Nevertheless, a limitation of this approach is its intrinsically lower sensitivity in the presence of small fat fractions that can be overcome with technical workarounds [use of small flip angles and fixed T1 values, as mentioned by Leporq et al. (24)]. Within these last two decades, 1H-MRS of the liver has proved to a be a valuable tool in metabolic research, in studies conducted on patients with increased risk for metabolic diseases. It can be used as a standard *in vivo* measurement for PDFF and shows high sensitivity to low lipid concentration when an appropriate number of acquisitions (typically 32) is averaged (56). In the proposed fasting intervention protocol, it provides a countermeasure to CSE-MRI measurement, to ensure the integrity of the PDFF results—especially at the limit of detection of the CSE-MRI method—as it is typically the case here, 4 months after the end of fasting.

Moreover, ^1^H-MRS is a unique way to explore and quantify non-invasively the amount of carnosine, a naturally occurring dipeptide recognized to multitask as an antioxidant acting against reactive oxidative (ROS) and reactive nitrogen species (RNS), as well as an anti-glycating agent to avoid intracellular accumulation of non-enzymatic glycation proteins leading to advanced glycation (AGE) or advanced lipoxidation end-products (ALE) (57). While found in excitable muscle and nervous tissues, it is synthesized by the carnosine synthetase from beta-alanine and L-histidine amino-acids. ^1^H-MRS is particularly valuable for carnosine quantification because of the metabolic instability of carnosine in human serum due to the presence of carnosinase. Some authors (58) have shown that deprivation of dietary beta-alanine (vegetarianism) seems to slightly affect muscle carnosine content negatively with no evidence of a disadvantageous aspect of the vegetarian diet on muscle adaptations. Based on carnosine monitoring, the same authors concluded that a vegetarian diet intervention did not negatively affect performance or muscle buffer capacity during a sprint training of 5 weeks. In addition to the pH-buffering and anti-oxidation stress role in skeletal muscle function, carnosine has been described as an anti-aging compound with a possible protective effect in neurological disorders, as well as a beneficial effect on diabetic complications (59).

In the studied volunteer, carnosine increased at the end of fasting (+20% compared to baseline) with a plateau effect and slight increase (+3%) within the few days after refeeding. After 1 month, carnosine level decreased to a level lower than baseline values (−32% compared to baseline) (Figure 4c). Note that absolute values need to be taken with caution as these estimations were made using T1 and T2 relaxation time from the literature. Also, other calibration strategies (i.e., water reference or external reference using solution with known carnosine concentration) to double check carnosine content might be worth considering as creatine content could be affected by nutrition (59–61). While these findings first require confirmation in a larger population, this change could be the signature of a reduced oxidative stress steady-state that have been illustrated in intermittent fasting protocols (62).

We found in our volunteer skeletal muscle lipid component changes of extra-myocyte lipids (EMCL) and intra-myocyte lipids (IMCL) components: from a near balanced amount of EMCL and IMCL at baseline, all lipids were increased, with a higher increase of IMCL (+133%) compared to EMCL (+53%), with an incomplete recovery after 1 month. These results are coherent with the results obtained by Wietek et al. (4) who explored the dynamics of the impact of food deprivation on the amount of intramuscular lipids, with a 4-fold increase of intramyocellular lipids (ratio lipid/creatine) in the quadriceps muscle. They also suggested that this augmentation of the intramyocellular lipid pool could be a marker of the long-term elevation of plasma FFA in the presence of low plasma insulin concentration during prolonged fasting.

Overall, the proposed imaging acquisition and analysis pipeline have the potential to refine our understanding on the impact of nutrition interventions such as periodic fasting, enabling to explore both spatial and temporal distribution and the relationship between bone marrow fat, liver fat, and muscle fat. This is an important area of investigation (18, 43, 62, 63) with, for instance, the recent evidence that saturated fatty acids may potentially exert lipotoxic effects on bone (47–49). When food supplies are very restricted, an adaptation occurs in the body that includes a metabolic shift from the use of glycogen stores in the liver and muscle cells to the mobilization of fatty acids in adipose cells and their conversion to ketones, an alternative substrate for cellular energy (64). Thus, fasting can enhance the ability of cells to remove molecular “garbage” (damaged proteins and organelles), which involves increased autophagy (65). A primary mechanism by which fasting can protect against tissue injury and disease is by activating adaptive cellular stress responses via hormesis-based processes (66–69). Having the possibility to non-invasively quantify thus monitor non-invasively obtained imaging biomarkers in each related organ is of very compelling interest to demonstrate the changes that indeed occur. It offers the possibility to review and correlate the whole landscapes of available biomarkers and understand their interplay. Note that while we used simple descriptors (mean values) of quantitative MR markers, more advanced radiomics markers could be of interest (70). Such analysis to obtain refined fingerprints derived from multiparametric maps could be helpful to further investigate the difference between subjects along the interventional diets as well as local and specific patterns in all investigated organs of interest.

## Limitations

This technical case report study is only a preliminary study. It is therefore mandatory to confirm its findings by future studies involving multiple subjects/groups, with the appropriate study design. The hypothetic protective effect of low-calorie diets and its temporal dynamics need, in particular, further investigations while controlling for the effect/modulation of gender, age, body type, and metabolic profiles.

## Conclusion

In conclusion, this single subject pilot study provides a good overview of the advanced MR imaging techniques that could be deployed on much larger studies aiming at quantitatively monitor fasting therapy by non-invasive measurements.

It would be of particular interest to confirm the observed trends in a larger cohort study while also investigating the persistence of the changes over a more extended period (e.g., 6 months to 1 year). The proposed advanced methodology to quantify the local and multi-organ impact of such a diet at multiple time points can now be transferred in clinical routine and combine with other omics evaluation to empower the detection of useful biomarkers in nutrition. We expect that this preliminary study will stimulate new insights and ideas for the future, promoting an efficient collaboration between bone and muscle biologists, endocrinologists.

Indeed, our modern industrialized societies have been exposed to a continuous supply of energy-dense processed food combined with rapid urbanization and the development of technologies that largely eliminated the need for physical exertion. This has led to increased risks of obesity and an enhanced prevalence of associated diseases, resulting in new social and economic burdens (1). A significant reduction in this burden will require the development of advanced and efficient methodologies together with population-wide interventions, including engaging healthcare providers and the development of not only substantial scientific evidence to support firm political commitments in turn favoring the involvement of individuals as the key stakeholders. Safety and efficacy of periodic or intermittent fasting should be established since these could play an essential role as a low-cost therapy.

## Author Contributions

PC and MV contributed to project development, MR data collection and management, data analysis, manuscript writing. BL contributed to project development, CSE-MRI data analysis, manuscript editing and discussion of obtained results. HR contributed to project development, MRS data analysis, manuscript editing and discussion of obtained results. BG contributed to project development, manuscript edition and discussion of obtained results. SD contributed to project development, description of fasting intervention and protocols, follow-up, and data collection at Buchinger Clinic during fasting, manuscript editing and discussion of obtained results. FW contributed to project development, description of fasting intervention and protocols, manuscript editing and discussion of obtained results.

### Conflict of Interest Statement

The authors declare that the research was conducted in the absence of any commercial or financial relationships that could be construed as a potential conflict of interest.
