# Supplementary material for: Chemical-Shift-Encoded Magnetic Resonance Imaging and Spectroscopy to Reveal Immediate and Long-Term Multi-Organs Composition Changes of a 14-Days Periodic Fasting Intervention: A Technological and Case Report
Source: Front Nutr. 2019 Mar 1;6:5. doi: 10.3389/fnut.2019.00005 (PMC6407435; doi:10.3389/fnut.2019.00005)
Supplement: Supplementary file 1 [file Table_1.docx]

Supplementary Table: Biological data before, end fasting, 1 week and 1 month after a 14-days Periodic Fasting intervention

| **Hematology data** | Norm | Units | Before | End fasting | 1 week after | 1Month after |
| --- | --- | --- | --- | --- | --- | --- |
| leukocytes | 3.9 - 9.8 | Tsd./µl | 5.4 | 4.5 | 3.89 | 5.5 |
| erythrocytes | 4.54 - 5.77 | Mio/µl | 5.13 | 5.34 | 5.33 | 5.2 |
| Hemoglobin | 13.5 - 17.5 | g/dl | 16.2 | 16.4 | 16.7 | 16.4 |
| hematocrit | 40.0 - 51.0 | % | 46.0 | 47.5 | 47 | 46.5 |
| MCV mean cell volume | 80.0 - 96.0 |  | 89.7 | 89.0 | 89 | 89.3 |
| MCH average cell volume | 28.0 - 33.0 | pg | 31.6 | 30.7 | 31 | 31 |
| MCHC mean cell volume | 33.0 - 37.0 | g/dl | 35.2 | 34.5 | 35.4 | 35 |
| platelets | 146 - 328 | Tsd./µl | 220 | 220 | 205 | 225 |
| Anisocytosis index | 12 -15 | % | 13 | 12 | 12.1 | 13 |
| **Coagulation** |  |  |  |  |  |  |
| Quick / INR Test | 0.90 - 1.26 | kA | 0.98 |  |  |  |
| Quick Test | 70 - 130 | % | 103 |  |  |  |
| PTT (Thromboplastin time) | 25 - 37 | sec | 31 |  |  |  |
| **Liver** |  |  |  |  |  |  |
| GOT | < 50 | U/l | 24 | 41 | 39 | 30 |
| GPT | < 50 | U/l | 33 | 49 | 31 | 31 |
| Gamma GT | < 60 | U/l | 30 | 19 | 15 | 25 |
| AP (Alk Phosphatase I.S.) | 40 - 130 | U/l | 61 | 54 | 58 | 60 |
| **Kidney** |  |  |  |  |  |  |
| Uric acid | < 7.0 | mg/dl | **7.0** | **11.0** | 7.29 | 7.0 |
| Urea | < 50.0 | mg/dl | 29.7 | 15.1 | 14 | 29 |
| Creatinine (Enzyme) | 0.58 - 1.23 | mg/dl | 0.96 | 0.89 | 1.01 | 0.97 |
| Thyroid |  |  |  |  |  |  |
| TSH (basal) | 0.30 - 4.0 | mE/l | 1.7 | 1.6 | 1.62 | 1.68 |
| **lipids** |  |  |  |  |  |  |
| Total cholesterol (T-Chol) | < 200 | mg/dl | **293** | **205** | 176 | 190 |
| HDL Cholesterol | 35 - 120 | mg/dl | 52 | 49 | 52 | 52 |
| Triglycerides (TG) | < 150 | mg/dl | **250** | 118 | 132 | 140 |
| LDL Cholesterol | < 160 | mg/dl | **181** | 126 | 97 | 118 |
| LDL / HDL | < 4.0 | kA | 3.5 | 2.6 | 1.87 | 2.26 |
| **Glucose** |  |  |  |  |  |  |
| Serum Glucose | 60 - 100 | mg/dl | 96 | 60 | 95 | 95 |
| HbA1c | < 39 | mmol/mol | 33 | 29 | 31 | 31 |
| glycated hemoglobin | < 5.7 | % | 5.2 | 4.8 | 5 | 5 |
| **Minerals / trace elements** |  |  |  |  |  |  |
| Sodium | 132 - 146 | mmol/l | 141 | 140 | 140 | 141 |
| Potassium | 3.5 - 5.1 | mmol/l | 4.9 | 4.5 | 4.5 | 4.7 |
| Calcium | 2.08 - 2.65 | mmol/l | 2.46 | 2.42 | 2.43 | 2.46 |
| Magnesium | 0.65 - 1.05 | mmol/l | 0.89 | 0.89 | 0.85 | 0.87 |
| **Immune system** |  |  |  |  |  |  |
| Sedimentation |  | mm/h | 2//4 | 2//4 | 2 | 2 |
| C-reactive Protein | < 5.0 | mg/l | 0.06 | 0.53 | <0.3 | 0.3 |
